# Motor Unit Number Estimation in Normal and Parkinsonism Model of Medial Gastrocnemius Muscle in Rats

**Published:** 2013

**Authors:** Effat Barghi, Margaret Gladden

**Affiliations:** 1 *Cellular and Molecular Biology Research Center (CMBRC), Babol University of Medical Sciences, Babol, Iran.*; 2 *Institute of Biomedical and Life Sciences, West Medical Building, University of Glasgow, Scotland,UK.*

**Keywords:** Motor unit number, amplitude of motor unit potentials, medial gastrocnemius muscle, electromyography, parkinson's disease

## Abstract

Motor units (MUs) reflect the function of the central nervous motor system. Thus, the estimated MU number is a good option to investigate the functional movement disorder in the Parkinson’s disease (PD). The purpose of this study was to compare the estimated MUs number in the medial gastrocnemius (MG) muscle of the normal rats and those with the parkinsonism. The MG muscle of two age-matched group (normal and parkinsonism) of anesthetized male, adult (154-304 days old) Wistar rats were studied after the insertion of electromyography (EMG) needles. The insertion activity and the MU recruitment (MUR), the strengths of mechanical involuntary contractions and the evoked spike potentials, were recorded. The means initial and the maximal amplitudes of the motor unit potentials (MUPs) were calculated for the estimated MUs number. The spinal cord at the L4-L6 was removed for pathological study. The parkinsonism MUPs trace showed irregular and low threshold discharge rate. The normal spikes trace, however, was different. Increased age was not associated with any increase in the MU number in the two groups. However, there was a significant correlation between the mentioned parameters and the insertion activity (r= -0.25, r= -0.177) and the MUR (r= 0.86, r= 0.248) in the normal and the parkinsonism groups, respectively. There was a correlation between the ages and mean MUP amplitude in the normal and the parkinsonism insertion activity (r =0.766, r =0.659) and the MUR (r =0.89, r = 0.4), respectively. Similarly, there was a correlation between the ages and maximal amplitudes in the normal and parkinsonism groups (r =0.53, r =0.42; r =0.86, r =0.248), respectively, *(p*<0.001). In the parkinsonism group, there was no significant correlation between the MUs number and the mean MUPs amplitudes in the insertion activity (r= 0.074, *p*= 0.088) and the MUR (r= 0.226, *p*=0.762). The spinal cord in the parkinsonism group showed degenerated nerve fibers and apoptosis in the degenerative nerve fibers and in the medium and large motor neurons with Lewy bodies and neurofibrillary tangles. The small ones, however, remained intact. The parkinsonism MUPs, compared to normal ones, have lower threshold and recruit less MUs. The apoptotic medium and large motor neurons with Lewy bodies contribute to the disuse of the relative MUs, while small ones remain intact.

Motor unit (MU) is the functional unit of the skeletal muscle that comprises a single motor neuron and muscle fibers which innervates them (1, 2). The function of recruited MUs number can make a typical force in the activation of skeletal muscles, but this activity depends on the combination of recruited MUs number (3-5). In addition, the activity of the upper levels of the central nervous motor system can change in the properties of the MUs even if the spinal cord is intact (6, 7). Spinal cord contains several motor neuron pools that relay the messages from the upper brain to the MUs (8, 9). The specific central motor processes affect the pools (10, 11). The manifestation of certain defective central nervous organs such as substantia nigra in Parkinson's disease (PD) which can interfere to MU activity and the electromyographic (EMG) technique will be able to record MU disorder (12, 13). PD is a progressive neurodegenerative disease, characterized by the presence of lewy body, neurofibrillary tangle,and senile plaques (14-16). PD had long been known as a sole motor system disorder, but later on was discovered to have an effect on the sensation, perception, and emotional functioning (17-19). 

Poppele (2001) suggested that apart from the motor system, the sensorimotor such as muscle spindle are also involved in PD (20). Bradykinesia in these patients is accompanied with resting tremor and muscle rigidity that the latter is secondary to the modulation of agonist and antagonist muscular tone (21-24). The aim of this study was to estimate normal and parkinsonism MUs number in the medial gastrocnemius (MG) muscle in Wistar rats.

## Materials and Methods

This experimental study was carried out on to compute MUs number in MG muscle in normal (n=9) and parkinsonism (n=9), adult (154-304 days old) male Wistar rats of the same age groups. The parkinsonism rats were treated with MPTP (Sigma, USA) 10 mg/kg, I.P (25, 26). Bradykinesia, tail rigidity, and resting tremor were the predomi-nant sings of PD used to enroll the rats in this study. The rats were anesthetized, using supplemental doses (10 mg/kg, I.P.) of pentobarbital sodium (Sigma, USA) 30 mg/kg, I.P, if necessary. The rats were checked for absence of pinch reflex. Their body temperature was maintained at 37°C by machine control (Harvard Apparatus Limited, USA). At the end of the experiment, the rats were killed through overdosing the anesthetic drug. The left leg’s MG muscle was dissected and prepared for direct insertion of the monopolar needle (steel, 0.1-mm-diameter of tip that covered 20-muscle fibers /mm^2^) of the EMG electrodes into the muscle(13, 27).

The EMG signals were recorded after the application of mild to moderate and severe motions of the recording electrodes (equal to 0.05, 0.1mv), considered as insertion activity at rest. Thereafter, two different mechanical involuntary contractions (equal to 0.5, 5mv) were studied on the same muscle. The first two produced voltage, before achieving the threshold as evoked muscle action potential, and the next two voltage evoked maximal amplitude of motor unit potential (MUP) due to motor unit recruitment (MUR).

There were 2-3s intervals between the two tests. The spike potentials were recorded using Power Lab set (ML 866, 4 channels, AD Instru-ments Co. Australia), which had sweep velocity of 10ms/div, sensitivity of 100 µv/div, and high filtration rate of 1 KHz. The spikes amplitudes (peak-to-peak) were measured, then the means initial (n=10 spikes of each age group) and maximal amplitudes of the MUPs were calculated. Finally the number of units was estimated by dividing these two parameters to each other (28, 29). Thereafter a L4-L6 laminectomy was performed; the spinal cord was removed, immersed in 10% formaldehyde solution, and transverse section was stained with congo red for pathological study.

**Statistical analysis**

Student *t*-test was used to compare amplitude spike potentials in both groups; the p-value <0.05 was considered statistically significant and Pearson correlations wase used to asses the relationship between age and MUPs parameters. 

## Results

In the last visit, the parkinsonism rats showed typical bradykinesia. If they were impelled to move, they resisted and were agitated at the beginning, they showed clumsiness while moving. Distinctive rigidity in the tail’s muscles and resting tremor were clearly visible. Their hair changed from soft to spiky. 

**Comparison of the normal and the parkinsonism MUPs traces**

**Fig 1:**
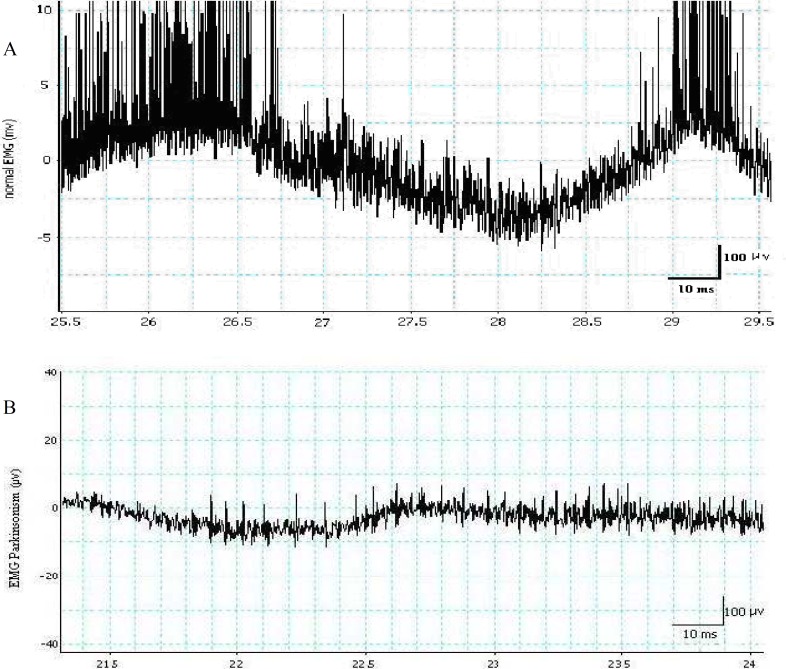
Trace ***A*** shows normal electromyogram (EMG), in the beginning, motor unit potentials recorded during mild to moderate and maximal involuntary contractions of medial gastrocnemius muscle, in the middle, discharged spike potentials by electrode motions, and the at the end, again evoked maximal spikes. Trace ***B*** depicts Parkinsonism EMG, initial evoked spike potentials recorded by electrode motions and recording sporadic with a few high spikes are due to muscle contractions

The two samples of the EMG spike potentials traces from the same age group, the normal and parkinsonism groups are shown in fig.1. The normal trace ***A*** shows two clusters of bursting high amplitude spike potentials produced by the MUR; short spikes due to insertion activity were evoked in the middle part. The normal MUPs appeared relatively constant with regular discharge rate. Trace ***B*** depicts an irregular discharge spike MUPs in parkinsonism with low threshold and very small amplitudes. The starting spike potentials were evoked by insertion activity and the sporadic spikes with high amplitudes were due to the MUR.

**Correlation between age and the MU number in normal and parkinsonism**

**Fig 2:**
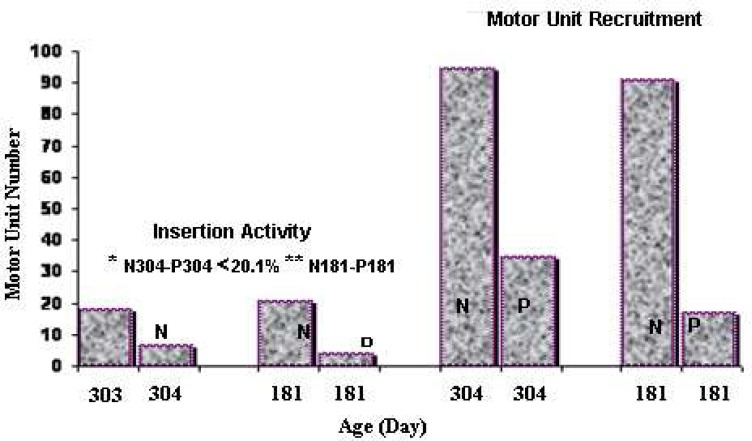
Comparison of different percentages of maximal and minimal of motor units number between the normal and the Parkinsonism, in the same age groups. N= normal, P = Parkinsonism, * = minimal values, ** = maximal values

Any increase in age is not exactly associated with increased MU numbers in both groups. The maximal difference of MUs number between the normal and the parkinsonism groups with 181 and 304-day-olds was 82.93% and the minimal diff-erence was 62.85%. These differences were more in the normal group than the parkinsonism group by the insertion activity testing (Table 1, Fig. 2). Whereas, for the foregoing ages of both groups the maximal difference of 81.33% and the minimal difference of 63.49% were found by the MUR test (Table 2, Fig.2). There were significant (*p*<0.001) inverse and weak correlation between the ages and the MUs number in the normal (r = -0.25) and parkinsonism (r= -0.177) groups by insertion activity. There was significant strong correlation in normal (r =0.86) rats and a weak one in the parkinsonism (r =0.248) group through the MUR. 

**Table1:** Results of motor unit potentials and MUN* of medial gastrocnemius muscle by the insertion activity

Age (day)	Normal Amplitude (μv) mean ± SD	Parkinsonism Amplitude (μv) mean ± SD	Normal Range of MUN*	Parkinsonism Range of MUN*
154	18.59 ± 2.22	3.16 ± 1.36	15-16	3-4
164	23.35 ±3.82	5.28 ± 1.85	17-18	3-4
181	22.61 ± 3.29	4.06 ± 1.21	20-21	5-6
213	22.40 ± 1.31	4.79 ± 1.42	17-18	4-5
231	24.45 ± 3.08	4.36 ± 1.47	18-19	4-5
244	22.02 ± 2.50	5.06 ± 1.44	16-17	3-4
262	23.94 ± 3.72	5.19 ± 0.98	18-19	4-5
284	25.73 ± 3.72	5.57 ± 1.73	17-18	4-5
304	26.28 ± 2.12	7.50± 2.5	17-18	6-7

*MUN=motor unit number

**Table 2:** Results of motor unit potentials and MUN* of medial gastrocnemius muscle by the MUR**.

Age (day)	Normal Amplitude (μv) mean ± SD	Parkinsonism Mplitude (μv) mean ± SD	Normal Range of MUN*	Parkinsonism Range of MUN*
154	151.99±6.61	17.91 ± 2.22	72-73	20-21
164	153.43±6.20	18.70 ± 2.31	75-76	19-20
181	131.41±4.86	17.23± 2.28	88-89	16-17
213	141. 6 141.6 ± 4.65	17.5 ± 1.96	83-84	17-18
231	184.49 ± 7.81	17.96 ± 4.73	90-91	17-18
244	189.40±5.79	21.33 ± 3.73	90-91	17-18
262	212.1± 6.77	17.81± 2.14	92-93	17-18
284	216.42±6.17	17.23 ± 3.08	92-93	21-22
304	215.59±5.19	21.47 ± 3.70	94-95	34-35

*MUN=motor unit number,

**=motor unit recruitment

**Correlation between the age and the amplitude of MUP**

**Fig 3:**
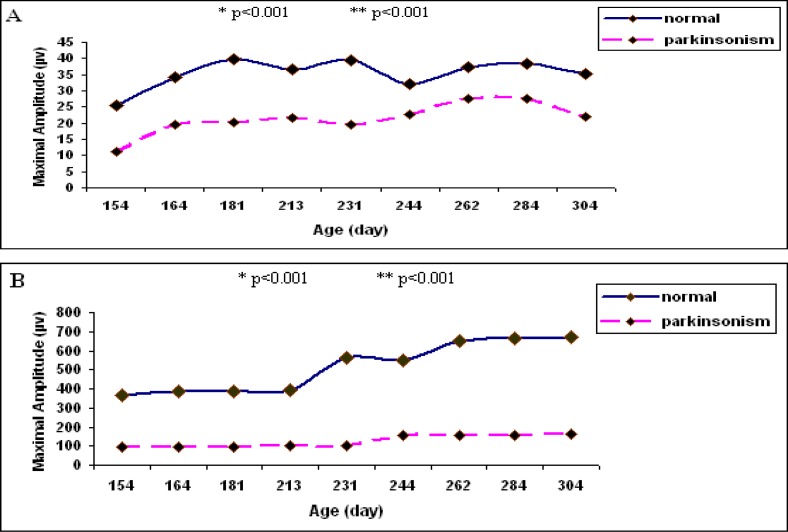
The comparison of the potentials of maximal amplitude motor unit between the normal and-- Parkinsonism during the application of insertion activity **(A)** and motor unit recruitment **(B)** in the same age groups. * and ** Relative amplitude differences Motor Unit Number in Normal and Parkinsonism

Any increase in age is associated with an increase in the means amplitude of the MUPs in both groups by the MUR, except for 2-cases of normal and 3-cases of parkinsonism which were different. The raised gradient manifested lesser nonconformity by the insertion activity compared to the MUR (Tables1 and 2). Despitethe asynchrony, there was a significant correlation between the age and the mean amplitude of the MUPs in the parkinsonism (r=0.659) and the normal ones (r=0.766) by insertion activity. The correlation was stronger in the normal group (r=0.89) compared to the parkinsonism (r=0.4) by the MUR. In addition, the correlation between increased ages and the maximal amplitude of the MUPs in the normal and the parkinsonism groups were unequal. However, the correlations between age and the maximal amplitude in the normal and the parkinsonism were significant following the insertion activity testing (Fig. 3A; r=0.53, r= 0.42) and the MUR (Fig. 3B; r= 0.946, r=0.72) f.

**Correlation of the MUs number and the amplitude of MUPs between the groups**

The correlation between the MUs number and means amplitude of the MUPs in the normal group (r= 0.428) by insertion activity and (r=0.682) by the MUR were significant (*p*<0.001). Such a correlation, however, was not reported in the parkinsonism group (r= 0.074, *p*=0.088) by insertion activity and (r=0.226, *p*=0.762) by the MUR. The MUPs data are shown in tables 1 and 2. Furthermore, the correlation between the MUs number and maximal amplitude of the MUPs in the normal (r= 0.63) and the parkinsonism (r=0.39) groups by insertion activity and by the MUR were significant (r=0.83; r=0.35) (*p*<0.001).

**Findings of pathological parkinsonism spinal cord**

**Fig 4:**
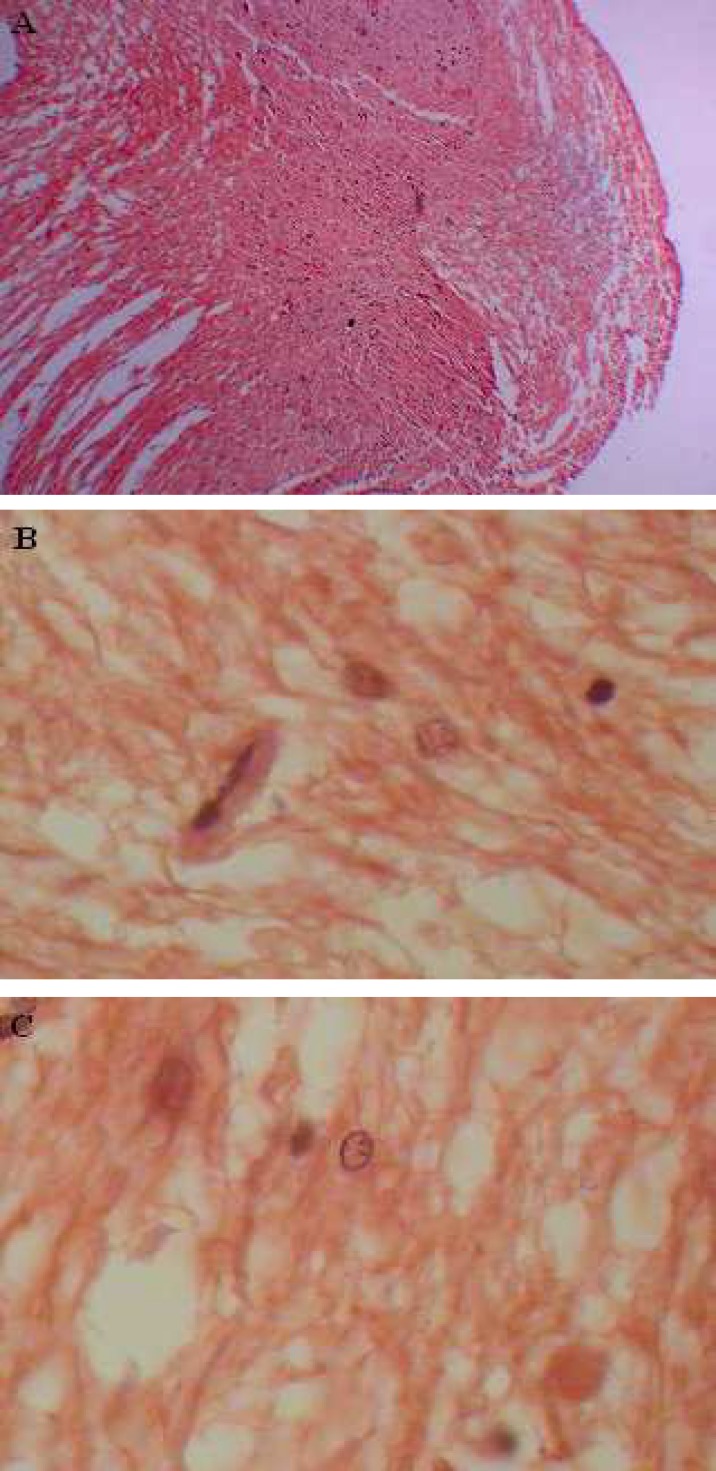
Parkinsonism model rat stained with CR. (A) A typical transverse section of Parkinsonism spinal cord. (B) The histology of Parkinsonism spinal cord, showing Neuro-fibrillary Tangle, chromatolysis of motor neurons, degenerative neurofibers, and Lewy bodies . (C) A typical Lewy body in necrosis motor neuron

The transverse section of parkinsonism spinal cord showed atypical apoptotic medium and large motor neurons under light microscopy. Lewy bodies and neurofibrillary tangles were also seen in the necrotic neurons. In addition, chromatolysis in both types of motor neurons and widespread degeneration neurofibers was observed. The small motor neurons were intact (Fig.4).

## Discussion

The present study shows that the normal and parkinsonism MUs of MG muscle have different perception and recruitment which depend on variable power stimuli reception and the next different discharge rate of spike potentials will be evoked. Petit (1990) reported that the MG muscle is a mixed muscle with three different types of fibers innervated with three different types of motor neurons located in spinal cord. They also showed that slow MUs are weaker than the two others (30). Luff (1992) reported a significant decrease in fast MUs and a small increase in slow MUs along with aging in normal MUPs in the MG muscle of the rats (31). The present study showed that any increase in age is not associated with an increase in the MUs number of normal and parkinsonism (r = -0.25, r = -0.177), respectively by the insertion activity. Despite the discordance between increasing age and MUs number, there was a significant correlation in normal group (r=0.86) but a weak correlation in parkinsonism group (r=0.248) by the MUR.The less recruitment of MUs number in the parkinsonism compared to the normal might be due to long delay and irregular long time stopping discharge of spike potentials. The comparisons of different percentages of MUs number between the insertion activity and the MUR trials on a pair of 181 and 304 days old from the normal and the parkinsonism groups, showed that these differences for a pair of 181 days old in the two types of trials were more than a pair of 304 days olds. According to these percentages, the severe affliction was for the young parkinsonism rather than the old. Meanwhile, in parkinsonism defective MUs activity without involuntary contraction showed 2.24% more damage than with involuntary contraction. Consequently, these findings could help interpreting the problem of the resting tremor in parkinsonism. The pathologic study of parkinsonism spinal cord shows apoptosis in the medium and large motor neurons with Lewy bodies. Certainly, the relevant MUs including fast-fatigue resistance and fast-fatigue have collapsed or became atrophied. Small motor neurons with relative oxidative MUs were intact; while Oishi (1992) found that slow MUs are more susceptible to atrophy (32). 

However, the medium and large motor neurons can not accurately perform sensible rece-iving messages tasks from the upper brain for relaying to the respective MUs. Some neurologists believed that impaired PD movement is caused secondary to the inadequate activation of excitatory circuits passing through from basal ganglia to spinal motor neurons and making insufficient facilitation to MUs (33). The current study ascertained that the slow motor neurons and relative MUs were intact but their functionality is not sufficient to dispel the weak contraction of parkinsonism MG muscle because low MUs number and low mean amplitude of the MUPs by the insertion activity (r =0.074,* P*=0.088) and the MUR (r =0.0226, *p*=0.762). These events showed the disability of large and medium motor neurons. Therefore, the MG muscle has lost harmonic functions which should therefore induce disorder movement in parkinsonism.

In conclusion, this study determined that the parkinsonism MUPs, compared to the normal ones, are unable to maintain high and constant discharge rates for extended periods of time, as MUPs have less recruited numbers, lower threshold, low mean values and shorter maximal amplitudes. Afflicted MUs to PD in younger was more than older MUs. More attention should be paid to the apoptotic medium and large motor neurons with Lewy bodies, but not for all three types of MUs and the default of MUs did not associate with the function of MG muscle. According to these findings, special factors for changing the spoil of MUs to healthy ones should be considered. 
